# Supplementary material for: GAPPadder: a sensitive approach for closing gaps on draft genomes with short sequence reads
Source: BMC Genomics. 2019 Jun 6;20(Suppl 5):426. doi: 10.1186/s12864-019-5703-4 (PMC6551238; doi:10.1186/s12864-019-5703-4)
Supplement: Supplementary file 1 — Data and commands used in the experiments. Data used in the experiments, parameters and commands used for running the tools. (PDF 196 kb) [file 12864_2019_5703_MOESM1_ESM.pdf]

GAPPadder: A Sensitive Approach for Closing Gaps on Draft Genomes with Short Sequence Reads

Chong Chu*, Xin Li and Yufeng Wu

1. Data and scaffolds used in the experiments

Table S1. Detailed information of the sequencing data

Species	Genome size (bp)	Coverage	Read length (bp)	Insert size
Staphylococcus aureus	~2,800,000	47	101	180
		46	37	3500
Human chrom 14	~107,043,718	34	101	180
		21	101	2283-2803
		2	76-101	35,295-35,318
Human whole genome	~3,300,000,000	48	101	300
		40	150	3000
Bed bug	~679,900,000	34.27	101	185
		12.16	101	367
		7.06	101	3000
Asian Sea bass	~668,481,366	43	101	500
		37	101	750

Table S2. Accession number of the sequencing data and assembly

Species	Reads Access No.	Assembly Access No.
Staphylococcus aureus	GAGE	GAGE (ALLPath-LG)
Human chrom 14		
Human whole genome	ERR194147	AEKP00000000.1
	ERP015131	
Bed bug	SRR1660254	JRLE00000000
	SRR1660256	
	SRR1660428	
Asian Sea bass	SRR3140997	LLXD00000000
	SRR3140998	

2. Command and parameter used to run each tool

For GapCloser and GapFiller, they all need a configuration files which indicates the basic information of the reads. So here we first show the commands used to run on different datasets, and then show the different configurations. Other parameters are kept the same for different datasets. For Sealer and GAPPadder, the parameters used are also kept the same for different datasets, and only need to change to related reads files when run the command.

2.1 Trim adapter for Mate-Paired reads

For the whole genome sequencing data, the mate paired end reads first need to trim the adapters. Nxtrim is used with the command:

/tools/NxTrim/nxtrim -1 reads_1.fastq -2 reads_2.fastq -O Insert_size_xx_reads –separate

2.2 Run GapCloser

./GapCloser -t 5 -l 101 -b XXX.config -a XXXX.scaffold.fa -o out.txt

2.3 Run GapFiller

perl /GapFiller_v1-10_linux-x86_64/GapFiller.pl -l lib.txt -s /chrom14/Assembly/Allpaths-LG/genome.scf.fasta -i 5 -b SingleDemo > temp_output.txt

2.4 Run Sealer

```
/abyss_install/bin/abyss-sealer -k90 -k80 -k70 -k60 -k50 -k40 -k30 -j 10 -B 1000 -P 10 -b 40G -o sealer_out -S genome.scf.fasta /All_reads/left.fastq /All_reads/right.fastq
```

2.5 Run GAPPadder

```
REF_FAI="XXX/Assembly/Allpaths-LG/genome.scf.fasta.fai"
GAP_POS="XXX/allpath_lg_gap_pos.txt"
N_JOBS=15
################################################
GAP_READS="gap_reads"
GAP_READS_ALIGNMENT="gap_reads_alignment"
GAP_READS_HIGH_QUALITY="gap_reads_high_quality"
if [ ! -d $GAP_READS ]
	then
	mkdir $GAP_READS
fi
if [ ! -d $GAP_READS_ALIGNMENT ]
	then
	mkdir $GAP_READS_ALIGNMENT
fi
if [ ! -d $GAP_READS_HIGH_QUALITY ]
	then
	mkdir $GAP_READS_HIGH_QUALITY
fi
###############################################
KMC_DIR="kmc_temp"
TEMP_DIR="temp"
KMER_DIR="kmers"
VELVET_DIR="velvet_temp"
BOTH_UNMAPPED="both_unmapped"
###############################################
if [ ! -d $KMC_DIR ]
	then
	mkdir $KMC_DIR
fi
if [ ! -d $TEMP_DIR ]
	then
	mkdir $TEMP_DIR
fi
```

```
if [ ! -d $KMER_DIR ]
	then
	mkdir $KMER_DIR
fi
if [ ! -d $KMER_DIR ]
	then
	mkdir $KMER_DIR
fi
if [ ! -d $VELVET_DIR ]
	then
	mkdir $VELVET_DIR
fi
if [ ! -d $BOTH_UNMAPPED ]
	then
	mkdir $BOTH_UNMAPPED
fi
################################################
python assembleGaps.py $REF_FAI $GAP_POS $N_JOBS
```

2.6 Configurations for GapCloser

2.6.1 Human chrom 14

```
[LIB]
avg_ins=180
reverse_seq=0
asm_flags=1
rank=1
q1=/chrom14/short_insert/frag_1.fastq
q2=/chrom14/short_insert/frag_2.fastq

[LIB]
avg_ins=3000
reverse_seq=1
asm_flags=2
rank=1
q1=/chrom14/long_insert_2k/shortjump_1.fastq
q2=/chrom14/long_insert_2k/shortjump_2.fastq

[LIB]
avg_ins=35000
```

reverse_seq=0
asm_flags=2
rank=1
q1=/chrom14/long_insert_30k/longjump_1.fastq
q2=/chrom14/long_insert_30k/longjump_2.fastq

2.6.2 Staphylococcus_aureus

[LIB]
avg_ins=180
reverse_seq=0
asm_flags=1
rank=1
q1=/Staphylococcus_aureus/short_insert/frag_1.fastq
q2=/Staphylococcus_aureus/short_insert/frag_2.fastq

[LIB]
avg_ins=3500
reverse_seq=1
asm_flags=2
rank=2
q1=/Staphylococcus_aureus/long_insert/shortjump_1.fastq
q2=/Staphylococcus_aureus/long_insert/shortjump_2.fastq

2.6.3 Human whole genome

[LIB]
avg_ins=3000
reverse_seq=0
asm_flags=1
rank=1
q1= /NA12878_mp_3k_35X/NA12878.mp.cor.1.fq
q2= /NA12878_mp_3k_35X/NA12878.mp.cor.2.fq

[LIB]
avg_ins=300
reverse_seq=0
asm_flags=1
rank=1
q1= /high_coverage_na12878/ERR194147_1.cor.fq

```
q2= /high_coverage_na12878/ERR194147_2.cor.fq
```

2.6.4 Bed bug

```
[LIB]
avg_ins=185
reverse_seq=0
asm_flags=4
rank=1
q1= /Bed_bug/short_insert/SRR1660254_1.fastq
q2= /Bed_bug/short_insert/SRR1660254_2.fastq

[LIB]
avg_ins=367
reverse_seq=0
asm_flags=4
rank=1
q1= /Bed_bug/short_jump/SRR1660256_1.fastq
q2= /Bed_bug/short_jump/SRR1660256_2.fastq

[LIB]
avg_ins=6000
reverse_seq=0
asm_flags=4
rank=1
q1= /Bed_bug/long_jump_nx/SRR1660428_1.fastq
q2= /Bed_bug/long_jump_nx/SRR1660428_2.fastq
```

2.6.5 Asian Sea Bass

```
[LIB]
avg_ins=500
reverse_seq=0
asm_flags=1
rank=1
q1= /Asian_Seabass/insert_size_500/SRR3140997_1.fastq
q2= /Asian_Seabass/insert_size_500/SRR3140997_2.fastq

[LIB]
avg_ins=750
reverse_seq=0
```

asm_flags=1
rank=1
q1= /Asian_Seabass/insert_size_750/SRR3140998_1.fastq
q2= /Asian_Seabass/insert_size_750/SRR3140998_2.fastq

2.7 Configuration file for GapFiller

2.7.1 Human Chrom 14

lib180 bwa /short_insert/frag_1.fastq /short_insert/frag_2.fastq 180 0.50 FR
lib3000 bwa /long_insert_2k/shortjump_1.fastq /long_insert_2k/shortjump_2.fastq 3000 0.50 RF
lib35000 bwa /long_insert_30k/longjump_1.fastq /long_insert_30k/longjump_2.fastq 35000 0.50 FR

2.7.2 Staphylococcus_aureus

lib180 bwa /short_insert/frag_1.fastq /short_insert/frag_2.fastq 180 0.50 FR
lib3500 bwa /long_insert/shortjump_1.fastq /long_insert/shortjump_2.fastq 3500 0.50 RF

2.7.3 Human whole genome

lib3000 bwa /NA12878.mp.cor.1.fq /NA12878.mp.cor.2.fq 3000 0.50 FR
lib300 bwa /ERR194147_1.cor.fq /ERR194147_2.cor.fq 300 0.50 FR

3. Percentage of the masked gap sequences for draft genome assembled by Velvet.

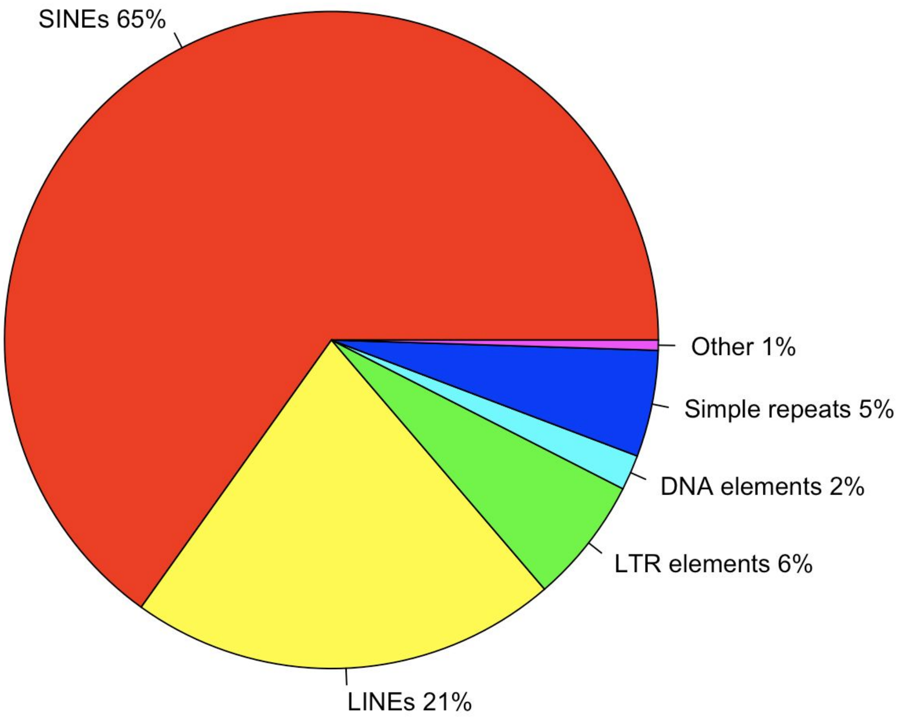

Fig S1. Percentage of the masked gap sequences of each type of repeats. Gaps are extracted from the Velvet assembled draft genome of human chromosome 14 that is released in GAGE. By aligning the flanking sequences to the human reference, we extract the gap sequences. We use RepeatMasker to get the types of repeats (e.g. LINE, SINE, LTR elements, and etc) of these gap sequences.

4. N50 statistic for the scaffolds before and after gap closing

We use the scripts released from GAGE (http://gage.cbcb.umd.edu/results/index.html) to calculate the N50 information for the human chromosome 14 scaffolds before and after gap closing. The detailed information are shown in Tab. S3.

Table S3. N50 statistic for the human chromosome 14 scaffolds before and after the gap closing

Type	# of contig	N50	# of corrected contig	Corrected N50
Before gap closing	1	81,646,936	9	3,471,817
After gap closing (GAPPadder)	1	81,778,652	8	3,534,119
After gap closing (GapCloser)	86	355,970	402	51,018
After gap closing (GapFiller)	1	81,672,699	8	3,471,492
After gap closing (Sealer)	1	81,606,259	9	3,317,972
